# Cost-effectiveness of lenvatinib plus pembrolizumab as the second-line treatment for advanced endometrial carcinoma

**DOI:** 10.1186/s12962-025-00711-y

**Published:** 2026-01-14

**Authors:** Szu-Ting Chiang, Chen-Han Chueh, Yu-Wen Wen, Shao-Chin Chiang, Wai-Hou Li, Yi-Wen Tsai

**Affiliations:** 1 Oncology Wards & Phase I Center, Department of Nursing, National Taiwan University Hospital, Taipei, Taiwan; 2 Herbert Wertheim School of Public Health and Human Longevity Science, University of California San Diego, San Diego, CA USA; 3 Department of Biomedical Science, College of Medicine, Chang Gung University, Taoyuan, Taiwan; 4 Department of Pharmacy, College of Pharmaceutical Sciences, National Yang Ming Chiao Tung University, Taipei, Taiwan; 5 The Center of Advanced Pharmacy Education, Koo Foundation Sun Yat-Sen Cancer Center, Taipei, Taiwan; 6 Gynecologic Robotic and Minimally Invasive Surgery Center, Shin Kong Wu Ho Su Memorial Hospital, Taipei, Taiwan; 7 Institute of Health and Welfare Policy, College of Medicine, National Yang Ming Chiao Tung University, Taipei, Taiwan; 8 Medical AI Development Center, Taipei Veterans General Hospital, Taipei, Taiwan

**Keywords:** Cost-effectiveness analysis, Endometrial carcinoma, Lenvatinib, Partitioned survival model, Pembrolizumab, Taiwan

## Abstract

**Background:**

The combination of lenvatinib and pembrolizumab (LP) for advanced or recurrent endometrial carcinoma has demonstrated improved survival in a clinical study and has been endorsed by the European Society for Medical Oncology. However, the LP regimen is currently not covered by Taiwan’s National Health Insurance (NHI). This study aimed to evaluate the cost-effectiveness of LP as a second-line treatment for advanced endometrial carcinoma (aEC) under Taiwan’s NHI.

**Methods:**

A 3-state partitioned survival model was used to analyze a hypothetical population of patients with aEC over 20 years. The overall and progression-free survival curves were obtained from the updated results of the Phase III trial 309/KEYNOTE-775 and extrapolated using a hybrid method. The pricing of the LP was based on the NHI fee schedule for other cancer treatments. Health state utilities and disutilities related to adverse events were obtained from the published literature. The willingness-to-pay (WTP) threshold was three times the GDP per capita in 2022 (NT$2,917,650). Quality-adjusted life-years (QALYs) and costs were discounted at 3%. Scenario analyses included LP price reductions and time horizon adjustments. Deterministic sensitivity, probabilistic sensitivity, and value of information analyses were performed to assess the uncertainty.

**Results:**

LP provided an incremental gain of 0.92 QALYs at an incremental cost of NT$2,929,046, resulting in an incremental cost-effectiveness ratio of NT$3,197,177, which exceeded the WTP threshold. The major factors causing uncertainty were the time horizon and cost of the LP.

**Conclusions:**

LP is not a cost-effective second-line therapy for aEC unless its price is reduced by more than 20%.

**Supplementary information:**

The online version contains supplementary material available at 10.1186/s12962-025-00711-y.

## Background

Endometrial cancer is a malignancy that affects women and originates in the inner uterine lining. Worldwide, it is the most common gynecological cancer and the sixth most prevalent cancer among women [1]. In Taiwan, the incidence of endometrial cancer has steadily increased from 11.11 to 17.03 per 100,000 individuals between 2011 and 2021, making it the fifth most prevalent cancer among women [2, 3]. As it progresses to advanced stages, the 5-year survival rate markedly decreases to 18% [4]. The first-line systemic treatment for advanced endometrial carcinoma (aEC) remains a platinum-based chemotherapy regimen comprising carboplatin and paclitaxel, and there is no standard of care for second-line chemotherapy due to limited efficacy and significant toxic effects [5–7].

Co-inhibition of vascular endothelial growth factor (VEGF) and programmed cell death protein 1 (PD-1) signaling, specifically, the combination of an immune checkpoint inhibitor (pembrolizumab) and a kinase inhibitor targeting angiogenesis and VEGF-mediated immune suppression (lenvatinib), demonstrated anti-tumor activity in patients with aEC in a Phase 2 trial [8]. This led to accelerated approval by the United States Food and Drug Administration in 2019 [9]. Full approval was granted in 2021 [10], supported by results from the Phase 3 Study 309/KEYNOTE-775 (NCT03517449) trial. This trial showed significant improvements in progression-free survival (PFS; HR: 0.56 [95% CI: 0.47–0.66]) and overall survival (OS; HR: 0.62 [95% CI: 0.51–0.75]) compared with chemotherapy [11]. This regimen is approved for women with aEC that had progressed after prior systemic therapy, who were ineligible for curative surgery or radiation, and who did not have high microsatellite instability (MSI-H) or mismatch repair-deficient (dMMR) status. In 2021, the European Commission approved this combination for patients with aEC who showed disease progression following prior platinum-containing therapy, regardless of their microsatellite or mismatch repair (MMR) status [12]. This recommendation has been supported by the UK’s National Institute for Health and Care Excellence (NICE) and the European Society for Medical Oncology (ESMO) [6, 13].

The combination of lenvatinib and pembrolizumab (LP) has been approved by the Taiwan Food and Drug Administration for this patient population. Lenvatinib and pembrolizumab have been reimbursed under Taiwan’s single-payer National Health Insurance (NHI) system for other indications since 2018 and 2019, respectively. Nevertheless, the LP regimen has not been reimbursed as a second-line treatment for aEC, with the high cost of combination therapy likely representing one of the considerations. Previous cost-effectiveness analyses (CEAs) have yielded mixed conclusions [11, 13–19], and their results are difficult to generalize to Taiwan due to differences in healthcare systems and direct medical costs. Therefore, this study aimed to evaluate the cost-effectiveness of the LP regimen as a second-line treatment for aEC within the framework of Taiwan’s NHI and to propose a reference price to address reimbursement concerns.

## Methods

### Cost-effectiveness analytical framework

**Fig. 1:**
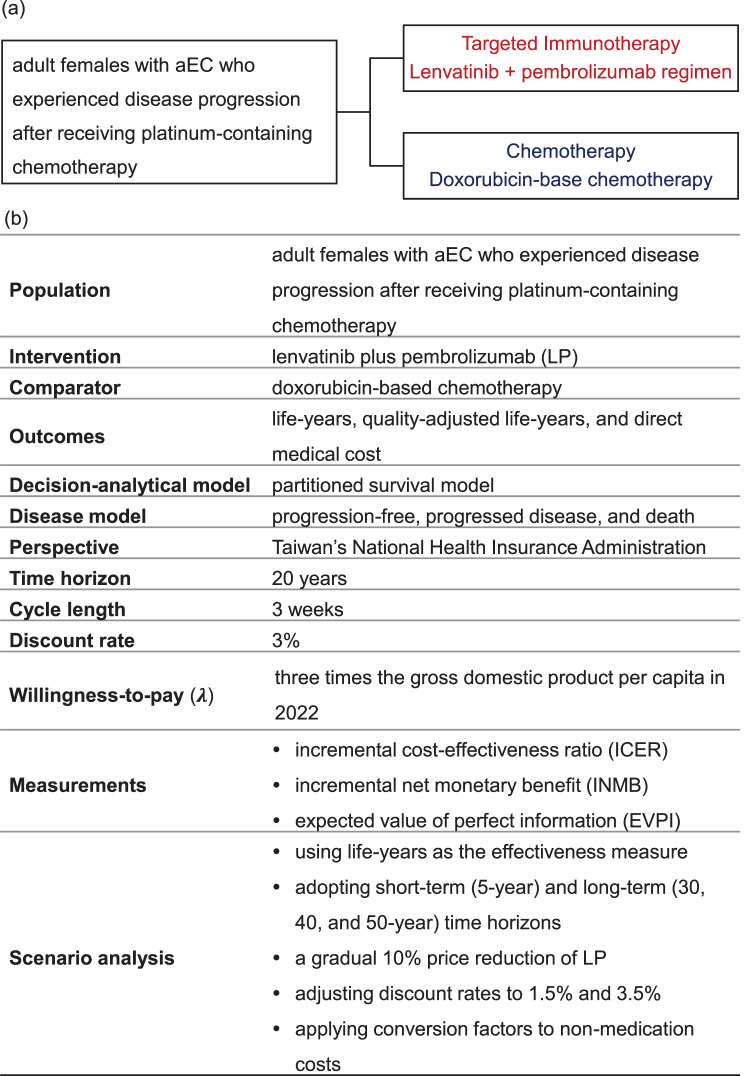
(**a**) Decision strategy: the intervention regimen provides LP, while the comparator offers doxorubicin-based chemotherapy; (**b**) cost-effectiveness analysis framework. aEC, advanced endometrial carcinoma

This study aimed to evaluate the cost-effectiveness of LP compared with doxorubicin in women with aEC who experienced disease progression after receiving platinum-containing chemotherapy from the perspective of Taiwan’s National Health Insurance Administration (NHIA). Figure 1a provides an overview of the CEA framework. We utilized a three-health state partitioned survival model (PSM) [20] and a 21-day cycle length to simulate cost and effectiveness over a 20-year time horizon. This duration was chosen based on the mean age of approximately 65 years in the 309/KEYNOTE-775 trial and the life expectancy at birth in Taiwan (2022), which is approximately 84 years [15, 21]. A hybrid method was employed to extrapolate the PFS and OS curves. Health outcomes and direct medical costs were discounted at an annual rate of 3% [22]. The willingness-to-pay (WTP) threshold was set at three times the gross domestic product (GDP) per capita in 2022 (NT$2,917,650), a common threshold adopted in oncology CEAs [23–25]. This study was analyzed using TreeAge Pro Healthcare (version 2024 R1; Williamstown, MA, USA; TreeAge Software, LLC).

### Target population

The simulated cohort mirrored the population of the Study 309/KEYNOTE-775 trial, consisting of women aged ≥18 years with advanced, recurrent, or metastatic endometrial carcinoma (excluding carcinosarcoma and sarcoma). Inclusion required disease progression after one prior platinum-based chemotherapy regimen, no prior exposure to VEGF- or PD-1–targeting agents, at least one measurable lesion per RECIST v1.1, and an Eastern Cooperative Oncology Group (ECOG) performance status of 0 or 1 [11]. The LP group included 411 patients with a median age of 64 years (range: 30–82); 15.8% had dMMR status, and 59.9% had an ECOG performance status of 1 [11]. Similarly, the chemotherapy group included 416 patients with a median age of 65 years (range: 35–86); 15.6% had dMMR status, and 57.9% had an ECOG performance status of 1 [11]. Detailed inclusion/exclusion criteria and baseline characteristics of the study cohort can be found in the primary publication [11].

### Intervention and comparator treatment protocols

The treatment strategies for the intervention and comparator groups followed the established protocol outlined in the 309/KEYNOTE-775 trial and adhered to Taiwan’s current reimbursement policy. In the intervention group, patients received 20 mg oral lenvatinib once daily in addition to 200 mg intravenous pembrolizumab every three weeks for up to 35 total doses [11]. In the comparator group, patients underwent chemotherapy with intravenous doxorubicin every three weeks (lifetime cumulative dose ≤500 mg/person) [11]. We assumed that patients received second-line systemic treatment until disease progression, followed by equal supportive care after disease progression.

### Decision-analytical model and effectiveness

We defined three distinct health states for economic evaluation: progression-free (PF), progressed disease (PD), and death. To estimate the proportion of membership in each health state per cycle, we utilized WebPlotDigitize (version 4.6) to extract data points from the Kaplan–Meier (KM) curves of PFS and OS in the 309/KEYNOTE-775 trial [15, 26]. The algorithm developed by Guyot et al. was implemented in the R software (version 4.2.2) to generate pseudo-individual patient data (IPD) [27]. We employed the IPDfromKM package in R to evaluate the precision of the OS and PFS plots derived from our reconstructed data points [28]. Six parametric models (exponential, Weibull, Gompertz, log-logistic, log-normal, and generalized gamma distributions) were fitted for PFS and OS. Model selection was based on visual assessment, Akaike information criterion, Bayesian information criterion, and expert opinion to determine the best-fit and the most appropriate extrapolated survival model (Table S1). The best-fit OS and PFS parametric models were identified as log-normal distributions for LP and log-logistic distributions for chemotherapy. Finally, a hybrid method was employed to establish the final PFS and OS curves, incorporating the KM curves within the trial period and the best-fit parametric survival model beyond the extrapolation period [29].

### Cost and adverse events

Table 1 presents the model parameters with point estimates and distributions for probabilistic sensitivity analysis (PSA). To estimate direct medical costs from real-world data, we used data from the Taiwan Cancer Registry, NHI Database (NHIDB), and National Death Certification Registry to assess the costs for the target population. From the Taiwan Cancer Registry dataset, we identified adult individuals with histologically confirmed aEC (ICD-O-3: 8263, 8380, 8382, 8570; ICD-9-CM: 182.0; ICD-10: C54.1) between January 1, 2011, and December 31, 2019, excluding cases with missing diagnosis dates. To identify patients undergoing second-line chemotherapy in the NHIDB, we selected women with aEC who initially received cisplatin- or carboplatin-based chemotherapy and later switched to doxorubicin-containing chemotherapy. Based on clinical expert consultation, a full course of second-line doxorubicin for aEC typically consists of six cycles. The PF state was defined from initiation of second-line treatment until the earliest occurrence of disease progression, death, or end of follow-up. Disease progression was identified by initiation of third-line chemotherapy or, among patients who did not complete six cycles, by discontinuation of second-line treatment. Discontinuation was defined as the expected date of the next treatment cycle (approximately one month) following the final doxorubicin prescription.

**Table 1:** Model parameters, baseline values, ranges, and distributions for sensitivity analyses

Parameters	Estimated value	DSA		PSA			References
		low	high	Distribution	Parameter 1	Parameter 2	
**Overall survival model**							
LP: log-normal	ln(mean) = 2.911	2.781	3.042	normal (μ, σ)	2.911	0.067	[15]
	ln(sd) = 1.245	1.138	1.262	normal (μ, σ)	1.245	0.057	[15]
Chemotherapy: log-logistic	shape = 1.683	1.538	1.841	normal (μ, σ)	1.683	0.077	[15]
	scale = 11.905	10.760	13.172	normal (μ, σ)	11.905	0.614	[15]
**Progression-free survival**							
LP: log- normal	ln(mean) = 2.002	1.883	2.121	normal (μ, σ)	2.002	0.061	[15]
	ln(sd) = 1.172	1.089	1.276	normal (μ, σ)	1.172	0.048	[15]
Chemotherapy: log-logistic	shape = 1.885	1.721	2.065	normal (μ, σ)	1.885	0.088	[15]
	scale = 3.956	3.597	4.351	normal (μ, σ)	3.956	0.192	[15]
**Risk for severe adverse events in lenvatinib plus pembrolizumab group**							
Hypertension	0.392	0.294	0.490	beta (α, β)	159	406	[15]
Diarrhea	0.081	0.061	0.101	beta (α, β)	33	406	[15]
Decrease appetite	0.076	0.057	0.095	beta (α, β)	31	406	[15]
Weight loss	0.108	0.081	0.135	beta (α, β)	44	406	[15]
Fatigue	0.054	0.041	0.068	beta (α, β)	22	406	[15]
Proteinuria	0.052	0.039	0.065	beta (α, β)	21	406	[15]
Anemia	0.069	0.052	0.086	beta (α, β)	28	406	[15]
Neutropenia	0.020	0.015	0.025	beta (α, β)	8	406	[15]
**Risk for severe adverse events in the chemotherapy group**							
Hypertension	0.026	0.020	0.033	beta (α, β)	10	388	[15]
Diarrhea	0.021	0.016	0.026	beta (α, β)	8	388	[15]
Decrease appetite	0.005	0.004	0.006	beta (α, β)	2	388	[15]
Weight loss	0.003	0.002	0.004	beta (α, β)	1	388	[15]
Fatigue	0.031	0.023	0.039	beta (α, β)	12	388	[15]
Proteinuria	0.003	0.002	0.004	beta (α, β)	1	388	[15]
Anemia	0.155	0.116	0.194	beta (α, β)	60	388	[15]
Neutropenia	0.260	0.195	0.325	beta (α, β)	101	388	[15]
**Utility**							
PF state	0.817	0.797	0.836	beta (α, β)	73.53	16.47	[30]
PD state	0.779	0.699	0.859	beta (α, β)	70.11	19.89	[30]
**Disutility**							
Hypertension	0.050	0.038	0.063	beta (α, β)	95	1804	[31]
Diarrhea	0.001	0.001	0.001	beta (α, β)	-	-	[32]
Decrease appetite	0.002	0.002	0.003	beta (α, β)	-	-	[32]
Fatigue	0.070	0.053	0.088	beta (α, β)	-	-	[32]
Anemia	0.073	0.055	0.091	beta (α, β)	93	1176	[31]
Neutropenia	0.460	0.345	0.575	beta (α, β)	54	63	[31]
**Medication cost (per cycle, NT$)**							
Lenvatinib	40,656	30,492.00	50,820.00	uniform (α, β)	1,452	2,420	NHI fee schedule
Pembrolizumab	98,668	74,001	123,335	uniform (α, β)	74,001	123,335	NHI fee schedule
Doxorubicin	3,779	2,834	4,724	gamma (μ, s)	3,779	167	Estimated from NHIDB
Other chemotherapy	2,999	2,249	3,749	gamma (μ, s)	2,999	671	Estimated from NHIDB
**Adverse event cost (per cycle, NT$)**							
Hypertension	28	21	35	gamma (μ, s)	28	4	Estimated from NHIDB
Diarrhea	25	19	31	gamma (μ, s)	25	11	Estimated from NHIDB
Decreased appetite	524	393	655	gamma (μ, s)	524	105	Estimated from NHIDB
Weight loss	562	422	703	gamma (μ, s)	562	121	Estimated from NHIDB
Anemia	674	506	843	gamma (μ, s)	674	115	Estimated from NHIDB
Neutropenia	2,544	1,908	3,180	gamma (μ, s)	2,544	564	Estimated from NHIDB
**Non-medication cost (per cycle, NT$)**							
PF state	22,149	16,612	27,686	gamma (μ, s)	22,149	2,971	Estimated from NHIDB
Supportive care	49,742	37,307	62,178	gamma (μ, s)	49,742	7,309	Estimated from NHIDB
**Conversion factor**	0.9	-	-	uniform (α, β)	0.8	1	Assumption

The costs are provided in 2022 NT dollars. DSA, deterministic sensitivity analysis; LP, lenvatinib plus pembrolizumab; NHI, National Health Insurance; NHIDB, National Health Insurance Database; NT$, New Taiwan dollar; PD, progressed disease; PF, progression-free; PSA, probabilistic sensitivity analysis

All costs in the PF and PD states were modeled using gamma distributions (Table 1). Cost parameters during the PF and PD states were estimated based on medical expenditures covered by the NHI. PF costs included medication expenses for second-line systemic therapy, adverse event (AE) management expenses, and non-medication medical costs. Non-medication costs encompass all NHI-covered fees incurred during inpatient and outpatient visits, such as diagnosis, drug administration, and laboratory examinations, that are unrelated to chemotherapy medication or AE management. As the Taiwan NHIA does not reimburse LP for aEC, we calculated the medication cost of LP based on its listing price for other cancers and the protocol of the 309/KEYNOTE-775 trial. Other costs, including medication costs for doxorubicin-based chemotherapy, non-medication costs, and supportive care costs, were estimated using NHI claims data for patients with aEC who failed first-line platinum-based chemotherapy and started second-line doxorubicin-based therapy. The average medication costs per cycle for LP and doxorubicin-based chemotherapy in second-line therapy were NT$139,324 and NT$6,778, respectively. The non-medication costs in the PF states were NT$22,149 per cycle for both treatments. Supportive care costs in the PD state were NT$49,742 per cycle for both treatment arms.

AEs were identified based on an incidence exceeding 5% and a severity greater than grade 3 in any treatment arm of the 309/KEYNOTE-775 trial [15]. These events included hypertension, diarrhea, decreased appetite, weight loss, neutropenia, anemia, fatigue, and proteinuria. The clinical management strategies for most AEs were based on information from the 309/KEYNOTE-775 trial and clinical experts. The cost associated with anemia was calculated based on the expenses related to packed red blood cell transfusions. We assumed that no medical expenses were incurred to manage the fatigue or proteinuria. AEs were assumed to have occurred during the first cycle of the PF state.

The NHIA employs a point-based fee system. In the base-case analysis, each point was assumed to be equivalent to NT$1 for the streamline calculations. For scenario analysis, a conversion factor of 0.9 (uniform distribution) was applied to non-medical costs to relax this assumption, considering that the average value of each point ranged between 0.8 and 1.

### Utility

Consistent with a previously published cost-effectiveness analysis [30], health state utilities for patients with aEC were assigned as 0.817 for PF, 0.779 for PD, and 0 for death, assuming that these parameters followed beta distributions. Disutility values for AEs were extracted from published literature [31, 32], and the proportion and disutility level of each AE are detailed in Table 1.

### Cost-effectiveness analyses

We calculated the incremental cost-effectiveness ratio (ICER) as the incremental cost per incremental quality-adjusted life-year (QALY) associated with LP compared with the doxorubicin-based chemotherapy regimen in Taiwan. The incremental net monetary benefit (INMB) was calculated as the net monetary benefit of LP compared with doxorubicin-based chemotherapy.

To ensure the robustness of our base-case results, we performed deterministic sensitivity analysis (DSA), PSA, and scenario analyses. In DSA, survival function parameters and health state-related utilities varied within 95% confidence intervals, and other parameters were adjusted by ±25% from baseline estimates. PSA involved a Monte Carlo simulation with 1,000 iterations to generate cost-effectiveness acceptability curves (CEAC) and calculate the expected value of perfect information (EVPI) to understand the analysis uncertainty. Scenario analyses explored the impact of different conditions, including using life-years as the effectiveness measure, adopting short-term (5-year) and long-term (30-, 40-, and 50-year) time horizons, implementing a gradual 10% price reduction of LP, adjusting discount rates, and applying conversion factors to non-medication costs.

### Model Validation

Model validation was conducted following the Assessment of the Validation Status of Health-Economic decision models [33]. Our decision-analytical model was aligned with the model used in the NICE report. Due to the unavailability of the original IPD from the trials, we chose a PSM instead of a Markov model. Two researchers independently reviewed the analytical decision models and programs to ensure accuracy.

### Ethics approval

This study was approved by the Institutional Review Board of the National Yang-Ming University, Taiwan (Approval No. YM111012E).

## Results

### Base-case and sensitivity analyses

Compared to doxorubicin-based chemotherapy, LP resulted in an incremental gain of 0.92 QALYs at an incremental cost of NT$2,929,046. This resulted in an ICER of NT$3,197,177 per QALY and a negative INMB of NT$244,808 (Table 2).

**Table 2:** Base-case cost-effectiveness results

Treatment strategy	Lenvatinib plus pembrolizumab	Doxorubicin-base chemotherapy	Incremental change
**Cost (NT$)**	**4,203,067**	**1,274,021**	**2,929,046**
PF state	2,815,232	277,882	2,537,350
PD state	1,387,835	996,139	391,696
**LYs**	**2.88**	**1.74**	**1.13**
PF state	1.27	0.59	0.68
PD state	1.60	1.15	0.45
**QALYs**	**2.29**	**1.37**	**0.92**
PF state	1.04	0.48	0.56
PD state	1.25	0.90	0.35
**ICER (NT$ per QALY)**			
Incremental cost per LY gained			**2,580,678**
Incremental cost per QALY gained			**3,197,177**
**INMB (NT$)**			
LY			**367,899**
QALY			**-244808**
**EVPI/person (NT$)**			**3,903**

The costs are provided in 2022 NT dollars. EVPI, expected value of perfect information; ICER, incremental cost-effectiveness ratio; INMB, incremental net monetary benefit; LY, life-year; PD, progressed disease; PF, progression-free; NT$, New Taiwan dollar; QALY, quality-adjusted life-year

**Fig. 2:**
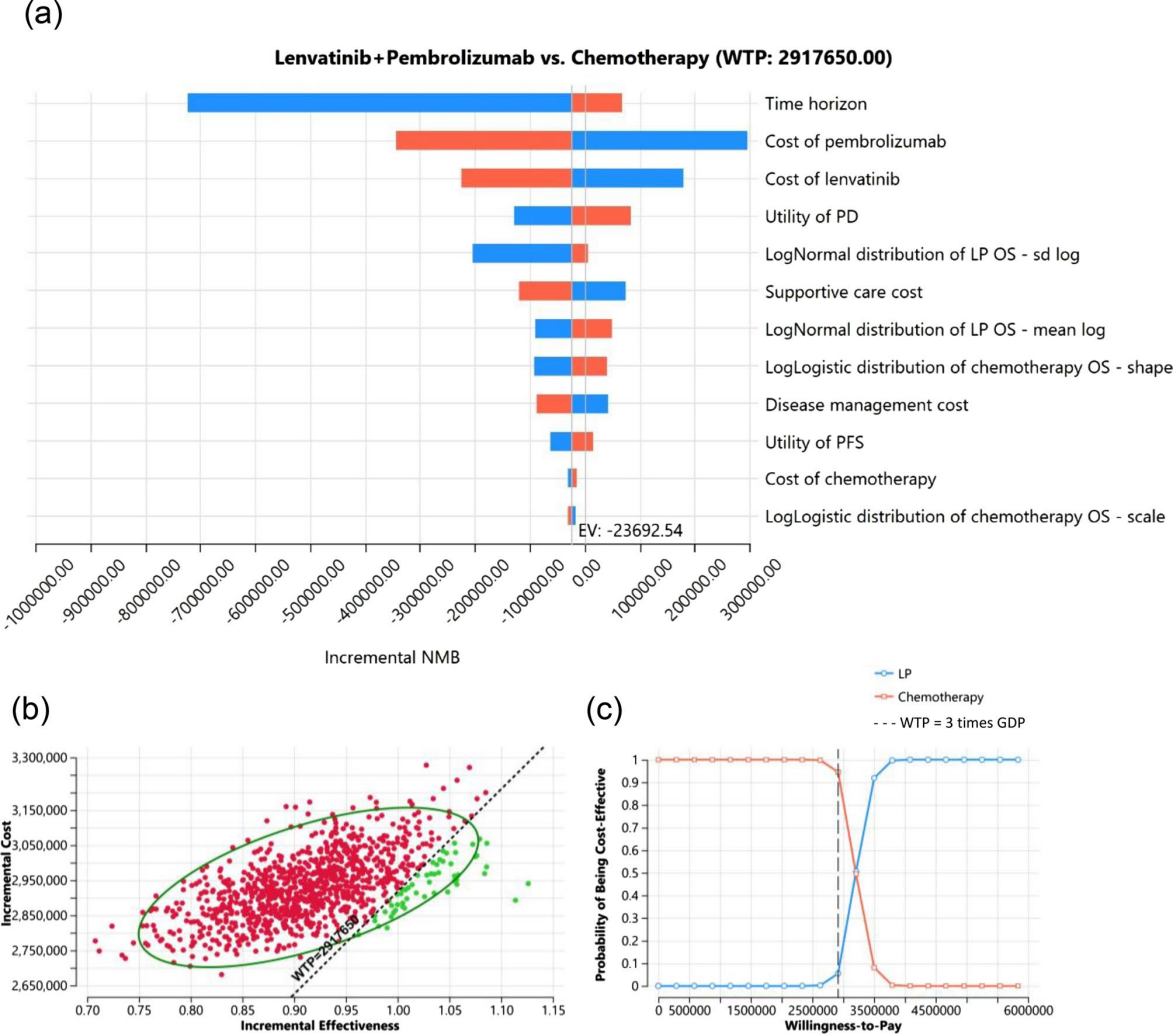
Deterministic sensitivity analysis and probabilistic sensitivity analysis results. (**a**) Tornado diagram of the top 12 parameters that cause uncertainty; (**b**) incremental cost-effectiveness plane for LP versus doxorubicin-based chemotherapy; (**c**) cost-effectiveness acceptance curve for LP versus doxorubicin-based chemotherapy. The costs are provided in 2022 NT dollars. GDP, gross domestic product; WTP, willingness-to-pay; PD, progressed disease; PF, progression-free; LogNormal distribution of LP OS – sd log, sdlog parameter of overall survival for lenvatinib plus pembrolizumab; LogNormal distribution of LP OS – mean log, mean log parameter of overall survival for lenvatinib plus pembrolizumab; LogLogistic distribution of chemotherapy OS – shape, the parameter of overall survival shape for chemotherapy; LogLogistic distribution of chemotherapy OS – scale, the parameter of overall survival scale for chemotherapy; LP, lenvatinib plus pembrolizumab

One-way sensitivity analysis revealed that the time horizon and medication cost of LP were the primary factors contributing to uncertainty (Fig. 2a). The results from 1,000 iterations of the Monte Carlo simulation are depicted in the cost-effectiveness cloud and CEAC in Figs. 2b and 2c, respectively. Compared with doxorubicin-based chemotherapy, LP demonstrated increased effectiveness with higher costs, resulting in a 5.5% probability of being deemed cost-effective at a WTP threshold of three times the GDP per capita per QALY gained. The EVPI (i.e., the opportunity cost associated with the LP regimen) was NT$3,903/person (Table 2).

### Scenario analysis

When the LP medication price was reduced by 20%, cost-effectiveness presented a positive INMB of NT$219,976. When life years were the outcome measure, the analysis indicated that compared to doxorubicin-based chemotherapy, LP had an increase of 1.13 life years, resulting in an ICER of NT$2,580,678 per life-year, with a 98.8% probability of being cost-effective. The base-case analysis results remained insensitive to other scenarios, including an adjusted time horizon, discount rate changes, and alternative conversion factors for non-medication costs (Table 3).

**Table 3:** Scenario analysis results: base case and probabilistic sensitivity analyses of all scenarios

Scenario	Base-case analysis		Probabilistic sensitivity analysis	
	ICER	INMB	Probability of Cost-effectiveness	EVPI/person
Base-case	3,197,177	−244,808	5.5%	3,903
Scenario				
Life years	2,580,678	367,899	98.8%	688
5-year time horizon	4,772,241	−939,589	0.0%	0
30-year time horizon	3,120,505	−200,293	13.6%	13,612
40-year time horizon	3,098,025	−184,445	16.8%	21,377
50-year time horizon	3,090,487	−159,898	18.2%	21,307
90% price of LP	2,943,511	−12,407	41.7%	50,512
80% price of LP	2,689,846	219,976	84.4%	13,349
70% price of LP	2,436,180	452,368	99.0%	683
3.5% discount rate	3,244,216	−295,692	2.9%	1,787
1.5% discount rate	3,056,187	−131,168	21.9%	24,867
Conversion factor − 0.9	3,125,741	−179,363	10.5%	8,190

The costs are provided in 2022 NT dollars. LP, lenvatinib plus pembrolizumab; ICER, incremental cost-effectiveness ratio; INMB, incremental net monetary benefit; EVPI, expected value of perfect information

## Discussion

### Principal findings

Our findings suggest that adopting the ESMO-recommended strategy of administering the LP regimen to all patients with aEC, irrespective of MMR status, would not be cost-effective from the perspective of Taiwan’s healthcare system. Specifically, when compared to the standard doxorubicin-based chemotherapy, the CEA results showed an ICER of NT$3,197,177 (WTP, NT$2,917,650) and a probability of 5.5%. Adjusting the time horizon did not alter these findings. However, a 20% price reduction in LP per cycle renders this regimen cost-effective (ICER, NT$2,689,846; probability, 84.4%) with low uncertainty.

### Comparisons with previous studies

Several CEAs have evaluated the LP regimen as a second-line therapy for all women with aEC, regardless of their MMR status [13, 14, 16, 17], yielding inconsistent conclusions. Two US studies consistently indicated a lack of cost-effectiveness, with variations in trial result versions and model designs [14, 17]. Notably, the latest US findings suggest that the LP regimen is cost-effective only under a substantial price reduction of 61.95% [17]. In the same study, the LP regimen was considered cost-effective from the perspective of Chinese payers [17].

Our study and those from NICE and Sweden utilized PSMs based on the latest findings from the 309/KEYNOTE-775 trial (released in 2023) [13, 15, 16, 34]. Our study reached a distinct conclusion regarding cost-effectiveness compared to the other two studies. Methodologically, our approach differs primarily in terms of the time horizon, survival functions, and parameters employed (Table S2). Notably, we extended our time horizon to 40 years in the scenario analysis with no change in the cost-effectiveness conclusion. While the other two studies analyzed the original IPD from the trial, we reconstructed the pseudo-IPD from the published KM curves. These three studies adopted different survival functions and extrapolation strategies based on their respective rationales. Nevertheless, we attribute the variance in conclusions, particularly regarding the cost-effectiveness, to differences in parameters, notably the cost parameters. Taiwan’s healthcare system remains low-cost under global budget control, where the incremental cost of innovative systemic therapy during the PF state dominates all other costs over the lifetime.

### Other findings and implications

At the end of the three-year follow-up, the probabilities of patients in the LP group remaining PF and alive were approximately 14% and 9% higher, respectively, than those in the chemotherapy group. In our 20-year simulation, compared to doxorubicin-based chemotherapy, LP achieved incremental gains of 0.68 LYs and 0.56 QALYs in the PF state and 0.45 LYs and 0.35 QALYs in the PD state. The incremental LYs/QALYs of the LP were primarily contributed by the PF state, aligning with the trial results. Specifically, the incremental LYs for the PF state of LP are 1.5 times higher than those for the PD state, highlighting LP’s effectiveness in prolonging PFS.

Considering the higher risk of AEs above grade 3 associated with the LP regimen than with pembrolizumab monotherapy (90.1% vs. 12%) [15, 35], future CEAs should evaluate second-line treatment strategies recommended by the National Comprehensive Cancer Network that allocate treatment (pembrolizumab monotherapy or LP) based on patients’ microsatellite instability (MSI)/MMR status [5]. Furthermore, exploring AE-related disutility among women with aEC is imperative to mitigate result uncertainty.

### Strengths and limitations

We used population-level real-world claims data to estimate costs, including AE-related management expenses, and integrated AE-related disutility into our analysis. This approach ensures that our results are more conservative and closely align with Taiwan’s real-world healthcare system, providing valuable cost-effectiveness evidence within the framework of universal health insurance that is particularly relevant to the Asian context. While this contribution extends beyond informing local reimbursement decisions, our study shares limitations common to model-based CEA studies [36–40], encompassing inherent modeling assumptions and challenges associated with synthesizing parameters from both clinical trials and real-world data.

A primary limitation stems from the lack of access to IPD from the pivotal trial. To address this, we employed a validated method to reconstruct pseudo-IPD [27, 28], which allowed us to estimate OS and PFS functions for our PSM. However, the absence of original IPD prevented us from employing a Markov model to cross-validate our findings, as doing so without individual data requires unverifiable assumptions regarding the post-progression survival function. Furthermore, PSMs possess inherent structural limitations; unlike Markov models that explicitly define state transitions, PSMs assume independence between OS and PFS functions. However, it is noteworthy that two recent studies with access to patient-level trial data also utilized PSMs for their analyses [16, 17], supporting the validity of our chosen approach.

Uncertainties also exist regarding outcome parameters and utility values. First, we extrapolated efficacy from a 43-month trial to a 20-year time horizon. This simulation may overlook long-term factors, such as delayed doxorubicin cardiotoxicity or the potential waning of pembrolizumab’s treatment effect, thereby affecting the accuracy of long-term outcomes. Second, due to the unavailability of specific utility data for certain health states, we relied on literature regarding pembrolizumab monotherapy for dMMR aEC, assuming utility levels were consistent between pembrolizumab monotherapy and the LP combination [30]. Similarly, disutility data for AEs were derived from external sources, under the assumption that these values are consistent across different cancer types.

In terms of cost estimation and data sources, reliance on NHI claims data introduced specific challenges. In the KEYNOTE-775 trial, 25% of the control group received paclitaxel; however, because Taiwan’s NHI does not cover paclitaxel as a second-line treatment for aEC, our study focused solely on patients receiving doxorubicin-based chemotherapy. We assumed doxorubicin’s efficacy was comparable to paclitaxel (as noted in the NICE report) and that doxorubicin-based regimens exhibited consistent efficacy and costs. Additionally, identifying eligible patients via administrative claims, using proxy measures such as medication termination or regimen changes, presents challenges. Costs in the comparator arm may be underestimated if patients receiving self-paid paclitaxel or LP were inadvertently included. Lastly, due to the lack of NHI coverage for the LP regimen in this indication, we were required to assume that the intervention and comparator arms incurred the same non-medication and AE-specific costs per cycle.

## Conclusions

In our base-case analysis, LP was a cost-ineffective second-line treatment for all women with aEC unless the medication cost reduction of the LP regimen exceeded 20%. Future studies should assess the utility and AE-related disutility in women with aEC receiving LP and evaluate the cost-effectiveness of LP within a treatment strategy tailored to the patient’s MSI/MMR status.

## Electronic supplementary material

Below is the link to the electronic supplementary material.

Supplementary Material 1

Supplementary Material 2

## Data Availability

The data supporting the findings of this study are available from the Ministry of Health and Welfare, Taiwan. However, restrictions apply to the availability of these data, which were used under the license for the current study and are not publicly available.

## Abbreviations

AE: Adverse event
aEC: Advanced endometrial carcinoma
CEA: Cost-effectiveness analysis
CEAC: Cost-effectiveness acceptability curve
dMMR: Mismatch repair-deficient
DSA: Deterministic sensitivity analysis
ECOG: Eastern Cooperative Oncology Group
ESMO: European Society for Medical Oncology
EVPI: Expected value of perfect information
GDP: Gross domestic product
ICER: Incremental cost-effectiveness ratio
INMB: Incremental net monetary benefit
IPD: Individual patient data
KM: Kaplan–Meier
LP: Combination of lenvatinib and pembrolizumab
MMR: Mismatch repair
MSI: Microsatellite instability
MSI-H: High microsatellite instability
NHI: National Health Insurance
NHIA: National Health Insurance Administration
NHIDB: National Health Insurance Database
NICE: National Institute for Health and Care Excellence
OS: Overall survival
PD: Progressed disease
PD-1: Programmed cell death protein 1
PF: Progression-free
PFS: Progression-free survival
PSA: Probability sensitivity analysis
PSM: Partitioned survival model
QALY: Quality-adjusted life year
VEGF: Vascular endothelial growth factor
WTP: Willingness-to-pay

## References

[1] Sung H, Ferlay J, Siegel RL, Laversanne M, Soerjomataram I, Jemal A, et al. Global cancer statistics, 2020: GLOBOCAN estimates of Incidence and Mortality worldwide for 36 cancers in 185 countries. CA Cancer J Clin. 2021;71:209–49. 10.3322/caac.21660. 33538338 10.3322/caac.21660

[2] Taiwan Cancer Registry Center. Top 10 cancers in Taiwan, Incidence and Mortality Rates for Top 10 cancers in Taiwan. 2011. https://twcr.tw/wp-content/uploads/2023/04/Top-10-cancers-in-Taiwan-2011.pdf. (accessed 10 April 2024).

[3] Taiwan Cancer Registry Center. Top 10 cancers in Taiwan, Incidence and Mortality Rates for Top 10 cancers in Taiwan, 2021. https://twcr.tw/wp-content/uploads/2024/02/Top-10-cancers-in-Taiwan-2021.pdf. (accessed 10 April 2024).

[4] American Cancer Society, Endometrial Cancer Early Detect Diagn Staging. 2024, 2024. https://www.cancer.org/content/dam/CRC/PDF/Public/8611.00.pdf. (accessed 10 April 2024).

[5] National Comprehensive cancer Network, uterine neoplasms, version 1. 2024, 2024. https://www.nccn.org/professionals/physician_gls/pdf/uterine.pdf. (accessed 4 February 2024).

[6] Oaknin A, Bosse TJ, Creutzberg CL, Giornelli G, Harter P, Joly F, et al. Endometrial cancer: ESMO clinical practice guideline for diagnosis, treatment and follow-up. Ann Oncol. 2022;33:860–77. 10.1016/j.annonc.2022.05.009. 35690222 10.1016/j.annonc.2022.05.009

[7] Taiwan Cooperative Oncology group, gynecological cancer clinical diagnosis and treatment guidelines. Miaoli County: Research Institute: National Health; 2011.

[8] Makker V, Rasco D, Vogelzang NJ, Brose MS, Cohn AL, Mier J, et al. Lenvatinib plus pembrolizumab in patients with advanced endometrial cancer: an interim analysis of a multicentre, open-label, single-arm, phase 2 trial. Lancet Oncol. 2019;20:711–18. 10.1016/S1470-2045(19)30020-8. 30922731 10.1016/S1470-2045(19)30020-8 PMC11686814

[9] Food and Drug Administration. Simultaneous review decisions for pembrolizumab plus lenvatinib in Australia. Canada and US. 2019. https://www.fda.gov/drugs/resources-information-approved-drugs/simultaneous-review-decisions-pembrolizumab-plus-lenvatinib-australia-canada-and-us. (accessed 6 March 2024).

[10] Food and Drug Administration, FDA grants regular approval to pembrolizumab and lenvatinib for advanced endometrial carcinoma, 2021. https://www.fda.gov/drugs/resources-information-approved-drugs/fda-grants-regular-approval-pembrolizumab-and-lenvatinib-advanced-endometrial-carcinoma. (accessed 6 March 2024).

[11] Makker V, Colombo N, Casado Herraez A, Santin AD, Colomba E, Miller DS, et al. Lenvatinib plus pembrolizumab for advanced endometrial cancer. N Engl J Med. 2022;386:437–48. 10.1056/NEJMoa2108330. 35045221 10.1056/NEJMoa2108330 PMC11651366

[12] European Medicines Agency, Assessment Report: Keytruda; 2021, 2021. https://www.ema.europa.eu/en/documents/variation-report/keytruda-h-c-003820-ii-0105-epar-assessment-report-variation_en.pdf. (accessed 6 March 2024).

[13] National Institute for Health and Care Excellence. Pembrolizumab with lenvatinib for previously treated advanced or recurrent endometrial cancer. 2023. 40163600

[14] Feng M, Chen Y, Yang Y, Li Q. Lenvatinib plus pembrolizumab vs. chemotherapy in pretreated patients with advanced endometrial cancer: a cost-effectiveness analysis. Front Public Health. 2022;10:881034. 10.3389/fpubh.2022.881034. 35619813 10.3389/fpubh.2022.881034 PMC9127138

[15] Makker V, Colombo N, Casado Herraez A, Monk BJ, Mackay H, Santin AD, et al. Lenvatinib plus pembrolizumab in Previously treated advanced endometrial cancer: updated efficacy and safety from the randomized phase III study 309/KEYNOTE-775. J Clin Oncol. 2023;41:2904–10. 10.1200/JCO.22.02152. 37058687 10.1200/JCO.22.02152 PMC10414727

[16] Ralph L, Young K, Upadhyay N, Prabhu VS, Ljungcrantz C, Massaad R, et al. Cost effectiveness of pembrolizumab plus lenvatinib compared with chemotherapy for treating previously treated advanced endometrial cancer in Sweden. J Med Econ. 2024;27:483–91. 10.1080/13696998.2024.2329022. 38470404 10.1080/13696998.2024.2329022

[17] Liao X, Wu Y, Lin D, Gu D, Luo S, Huang X, et al. Lenvatinib plus pembrolizumab in the patients with advanced previously treated endometrial cancer: a cost-effectiveness analysis in the United States and in China. J Obstet Gynaecol Res. 2024;50:881–89. 10.1111/jog.15910. 38485235 10.1111/jog.15910

[18] Dioun S, Chen L, De Meritens AB, St Clair CM, Hou JY, Khoury-Collado F, et al. Cost-effectiveness of lenvatinib plus pembrolizumab versus chemotherapy for recurrent mismatch repair-proficient endometrial cancer after platinum-based therapy. Gynecol Oncol. 2024;182:70–74. 10.1016/j.ygyno.2023.12.027. 38262241 10.1016/j.ygyno.2023.12.027

[19] Zheng Z, Yang L, Xu S, Zhu H, Cai H. Cost-effectiveness analysis of lenvatinib plus pembrolizumab compared with chemotherapy for patients with previously treated mismatch repair proficient advanced endometrial cancer in China. Front Pharmacol. 2022;13:944931. 10.3389/fphar.2022.944931. 36249813 10.3389/fphar.2022.944931 PMC9561308

[20] Woods BS, Sideris E, Palmer S, Latimer N, Soares M. Partitioned survival and state transition models for healthcare decision making in Oncology: where are we Now? Value Health. 2020;23:1613–21. 10.1016/j.jval.2020.08.2094. 33248517 10.1016/j.jval.2020.08.2094

[21] National Development Council. Life expectancy at birth. 2024. https://pop-proj.ndc.gov.tw/main_en/Custom_Fast_Statistics_Search.aspx?n=138%26sms=0%26d=H01%26m=74. (accessed 16 July 2024).

[22] Center for Drug Evaluation. Taiwan. Methodology Guide for health technology assessment. 2014. https://www.taspor.org.tw/upload/20150609113558.pdf. (accessed 28 June 2022).

[23] Robinson LA, Hammitt JK, Chang AY, Resch S. Understanding and improving the one and three times GDP per capita cost-effectiveness thresholds. Health Policy Plan. 2017;32:141–45. 10.1093/heapol/czw096. 27452949 10.1093/heapol/czw096

[24] World Health Organization. Reducing risks, promoting healthy life. World Health Rep. 2002;2002.

[25] National Statistics. https://eng.stat.gov.tw/cp.aspx?n=2334. (accessed 20 February 2024).

[26] Rohatgi A, WebPlotDigitizer. 2023. https://apps.automeris.io/wpd/. (accessed 30 June 2022).

[27] Guyot P, Ades AE, Ouwens MJ, Welton NJ. Enhanced secondary analysis of survival data: reconstructing the data from published Kaplan-Meier survival curves. BMC Med Res Methodol. 2012;12:9. 10.1186/1471-2288-12-9. 22297116 10.1186/1471-2288-12-9 PMC3313891

[28] Liu N, Zhou Y, Lee JJ. IPDfromKM: reconstruct individual patient data from published Kaplan-Meier survival curves. BMC Med Res Methodol. 2021;21:111. 10.1186/s12874-021-01308-8. 34074267 10.1186/s12874-021-01308-8 PMC8168323

[29] Latimer NR, Adler AI. Extrapolation beyond the end of trials to estimate long term survival and cost effectiveness. BMJ Med. 2022;1:e000094. 10.1136/bmjmed-2021-000094. 36936578 10.1136/bmjmed-2021-000094 PMC9951371

[30] Thurgar E, Gouldson M, Matthijsse S, Amonkar M, Marinello P, Upadhyay N, et al. Cost-effectiveness of pembrolizumab compared with chemotherapy in the US for women with previously treated deficient mismatch repair or high microsatellite instability unresectable or metastatic endometrial cancer. J Med Econ. 2021;24:675–88. 10.1080/13696998.2021.1917140. 33866938 10.1080/13696998.2021.1917140

[31] Wan X, Luo X, Tan C, Zeng X, Zhang Y, Peng L. First-line atezolizumab in addition to bevacizumab plus chemotherapy for metastatic, nonsquamous non-small cell lung cancer: a United states-based cost-effectiveness analysis. Cancer. 2019;125:3526–34. 10.1002/cncr.32368. 31287562 10.1002/cncr.32368

[32] de Groot S, Redekop WK, Versteegh MM, Sleijfer S, Oosterwijk E, Kiemeney L, et al. Health-related quality of life and its determinants in patients with metastatic renal cell carcinoma. Qual Life Res. 2018;27:115–24. 10.1007/s11136-017-1704-4. 28917029 10.1007/s11136-017-1704-4 PMC5770482

[33] Vemer P, Corro Ramos I, van Voorn GA, Al MJ, Feenstra TL. AdViSHE: a Validation-assessment tool of health-economic models for decision makers and model users. Pharmacoeconomics. 2016;34:349–61. 10.1007/s40273-015-0327-2. 26660529 10.1007/s40273-015-0327-2 PMC4796331

[34] National Institute for Health and Care Excellence. Pembrolizumab with lenvatinib for previously treated advanced, metastatic or recurrent endometrial cancer, single technol. Appraisal. 2023https://www.nice.org.uk/guidance/ta904/evidence/appraisal-consultation-committee-papers-pdf-13070962189) [ID3811], (accessed 10 April 2024), *Committee Papers*. 40163600

[35] O’Malley DM, Bariani GM, Cassier PA, Marabelle A, Hansen AR, De Jesus Acosta A, et al. Pembrolizumab in patients with microsatellite instability-high advanced endometrial cancer: results from the KEYNOTE-158 study. J Clin Oncol. 2022;40:752–61. 10.1200/JCO.21.01874. 34990208 10.1200/JCO.21.01874 PMC8887941

[36] Chueh CH, Tsai YW, Chen ZR, Shiu MN, Wen YW, Chiang NJ. Cost-effectiveness analysis of a new second-line treatment regimen for advanced intrahepatic cholangiocarcinoma: biomarker-driven targeted therapy of pemigatinib versus 5-FU chemotherapy. Pharmacoeconomics. 2023;41:307–19. 10.1007/s40273-022-01227-6. 36575331 10.1007/s40273-022-01227-6

[37] Chen ZR, Chueh CH, Chiang NJ, Tsai YW. Cost-effectiveness of pemigatinib as a second-line treatment for advanced intrahepatic cholangiocarcinoma with fibroblast growth factor receptor 2 fusions in Taiwan: from the evidence of the phase II trial and the perspective of Taiwan’s National Health Insurance Administration. Cost Eff Resour Alloc. 2023;21:61. 10.1186/s12962-023-00473-5. 37697368 10.1186/s12962-023-00473-5 PMC10496386

[38] Chen KA, Huang WM, Chen EY, Ho PK, Chueh CH, Wen YW, et al. Cost-effectiveness of ivosidenib versus chemotherapy for previously treated IDH1-mutant advanced intrahepatic cholangiocarcinoma in Taiwan. BMC Cancer. 2024;24:622. 10.1186/s12885-024-12362-y. 38778261 10.1186/s12885-024-12362-y PMC11110281

[39] Chueh CH, Huang WM, Hong MY, Tsai YW, Chiang NJ, Chen HL. The cost-effectiveness of sugemalimab plus CAPOX in treating advanced gastric cancer: analysis from the GEMSTONE-303 trial. Cancers. 2025;17(19):3171. 10.3390/cancers17193171. 41097699 10.3390/cancers17193171 PMC12523858

[40] Chen HL, Chueh CH, Huang WM, Chan SH, Cheng HH, Chiang SC, et al. Modeling brain metastases in cost effectiveness analysis of atezolizumab for extensive stage small cell lung cancer. Sci Rep. 2025;15:39298. 10.1038/s41598-025-22966-4. 41214083 10.1038/s41598-025-22966-4 PMC12603174

