# Supplementary material for: Cost-effectiveness of lenvatinib plus pembrolizumab as the second-line treatment for advanced endometrial carcinoma
Source: Cost Eff Resour Alloc. 2026 Jan 14;24:42. doi: 10.1186/s12962-025-00711-y (PMC12969905; doi:10.1186/s12962-025-00711-y)
Supplement: Supplementary file 1 — Supplementary Material 1 [file 12962_2025_711_MOESM1_ESM.docx]

Table S1 AIC and BIC values in each survival model

| Regimen | Distribution | Progression-free survival | | Overall survival | |
| --- | --- | --- | --- | --- | --- |
|  |  | AIC | BIC | AIC | BIC |
| Lenvatinib + pembrolizumab | Exponential | 2318.151 | 2322.169 | 2371.076 | 2375.094 |
|  | Weibull | 2318.096 | 2326.133 | 2370.947 | 2378.984 |
|  | Log-normal | 2255.009 | 2263.046 | 2356.91 | 2364.947 |
|  | Log-logistic | 2255.834 | 2263.871 | 2357.411 | 2365.448 |
|  | Gompertz | 2288.431 | 2296.468 | 2372.712 | 2380.75 |
|  | Generalized gamma | 2262.942 | 2274.998 | 2358.288 | 2370.344 |
| Chemotherapy | Exponential | 1749.604 | 1753.634 | 2567.289 | 2571.319 |
|  | Weibull | 1744.865 | 1752.926 | 2557.576 | 2565.638 |
|  | Log-normal | 1684.87 | 1692.931 | 2533.263 | 2541.324 |
|  | Log-logistic | 1672.085 | 1680.146 | 2529.4 | 2537.461 |
|  | Gompertz | 1746.148 | 1754.209 | 2569.116 | 2577.177 |
|  | Generalized gamma | 1688.175 | 1700.268 | 2534.311 | 2546.403 |
