# Supplementary material for: Cost-effectiveness of lenvatinib plus pembrolizumab as the second-line treatment for advanced endometrial carcinoma
Source: Cost Eff Resour Alloc. 2026 Jan 14;24:42. doi: 10.1186/s12962-025-00711-y (PMC12969905; doi:10.1186/s12962-025-00711-y)
Supplement: Supplementary file 2 — Supplementary Material 2 [file 12962_2025_711_MOESM2_ESM.docx]

Table S2 Comparisons with NICE reports and Swedish CEA

| Perspective | **U.K. payer perspective**^1,2^ | | **Swedish healthcare perspective**^3^ | | **Taiwan’s NHIA** | |
| --- | --- | --- | --- | --- | --- | --- |
| **Population** | aEC in adults who have disease progression on or following prior treatment with a platinum-containing therapy in any setting and who are not candidates for curative surgery or radiation | | adult females with aEC with disease progression on or following prior treatment with a platinum-containing therapy in any setting, and who are not candidates for curative surgery or radiation | | adult females with histologically or cytologically confirmed aEC who experienced disease progression following first-line platinum-based systemic therapy | |
| **Intervention** | LP | | LP | | LP | |
| **Comparator** | TPC (paclitaxel or doxorubicin) | | TPC (paclitaxel or liposomal doxorubicin) | | doxorubicin-base chemotherapy | |
| **Decision analytical model** | partitioned survival models | | partitioned survival models | | partitioned survival models | |
| Disease status | PF, PD, Death | | PF, PD, Death | | PF, PD, Death | |
| Cycle length | 1 weeks | | 1 weeks | | 3 weeks | |
| Time horizon | 40 years | | 40 years | | 20 years | |
| Discount rate (annual) | 3.5% | | 3% | | 3% | |
| WTP threshold (λ) | N/A | | SEK1,000,000 | | NT$2,925,582 | |
| **Parameters and sources** | | | | | | |
| Efficacy data source | 309/KEYNOTE-775 trial original individual patient-level data | | 309/KEYNOTE-775 trial original individual patient-level data | | 309/KEYNOTE-775 trial reconstructed pseudo-individual patient data | |
| Extrapolation strategy | ．fit six parametric survival models ．one-knot spline model | | ．one- and two-piece methods | | ．fit six parametric survival models ．Hybrid method | |
| Survival function | ．OS model:  - LP: KM (26 weeks) + log-logistic  - ChT: KM (26 weeks) + exponential ．PFS model:   - LP: KM (10 weeks) + log-logistic  - ChT: KM (10 weeks) + log-logistic ．TOT model: generalized gamma | | ．OS model:  - LP: KM (26 weeks) + log-logistic  - ChT: log-normal (treatment switch adjustment HR, 0.6) ．PFS model:   - LP: KM (10 weeks) + generalized gamma  - ChT: KM (10 weeks) + generalized gamma ．TOT model: generalized gamma | | ．OS model  - LP: log-normal  - ChT: log-logistic ．PFS model  - LP: log-normal  - ChT: log-logistic | |
| Cost & AE cost source | BNF, eMIT, NHS reference costs, Unit Costs of Health and Social Care | | Swedish Dental and Pharmaceutical Benefits Agency drug–cost database and the Apoteket, Southern Healthcare Region, TLV appraisal, and literature review. | | NHI fee schedule & NHIDB | |
| Cost | ．drug acquisition costs ．drug administration costs ．health state resource use costs ．AE costs ．cost of testing ．subsequent treatment costs ．end-of-life care costs | | ．drug costs ．administration cost  ．health state resource use cost ．AE costs ．subsequent treatment costs ．end-of-life costs | | ．drug costs ．health state non-medication cost (include all NHIA-covered expenditures, such as administration cost, testing cost, inpatient visit cost, outpatient visit cost, and so on). ．AE costs | |
| AE costs | separate cost for greater than grade 3 AEs and more than 5% incidence of patients in 309/KEYNOTE-775 (including hypertension, diarrhea, anemia, Asthenia, fatigue, neutropenia, febrile neutropenia, and leukopenia). | | a specific cost for grade 3+ AEs and more than 3% of patients in 309/KEYNOTE-775 for LP and ChT groups. | | separate cost for incidence exceeding 5% and severity greater than grade 3 in any treatment arm of the 309/KEYNOTE-775 trial (including hypertension, diarrhea, decreased appetite, weight decrease, anemia, and neutropenia). | |
| Utility & disutility source | 309/KEYNOTE-775 trial EQ-5D data | | 309/KEYNOTE-775 trial EQ-5D data | | literature review | |
| Utility | time-varying utility | | time-varying utility | | health state utility values (from aEC) | |
| Disutility | a specific disutility value when experiencing severity greater than grade 3 AEs | | a specific disutility value when experiencing severity greater than grade 3 AEs | | separate value for incidence exceeding 5% and severity greater than grade 3 in any arm of the 309/KEYNOTE-775 trial (including hypertension, diarrhea, decreased appetite, fatigue, anemia, and neutropenia)  The disutility values were obtained from RCC and NSCLC. | |
| **Sensitivity analysis** |  | |  | |  | |
| OWSA | 10 parameters | | 10 parameters | | all parameters | |
| Scenario | ．time horizon ．1.5% annual discount rate ．mixed chemotherapy setting for comparator ．treatment dosing and duration  - paclitaxel: maximum duration of 6 months  - doxorubicin: no maximum dosing rule  - pembrolizumab dosing: 400mg Q6W  - lenvatinib weekly dosing: full 20 mg dose  - TOT: Weibull (both arms)  - TOT can not exceed PFS (both arms)  - TOT: directly based on full KM (pembrolizumab and TPC) ．efficacy assumptions  - OS: KM used for first 52 weeks  - PFS: LP/TPC 10-week KM + log-normal  - PFS: KM used for first 37 weeks  - PFS: LP/TPC independent one-piece log-logistic ．utility input  - use health state utility values  - use TTD utility model but exclude AE decrement  - utility: age-adjusted utility, No ．cost inputs  - Use Caelyx cost for doxorubicin  - Exclude AE costs  - assume there is vial sharing (no wastage)  - exclude subsequent treatment costs  - alternative distribution of subsequent treatments (ECHO) | | ．time horizon - 30 year ．discount rate  - 0% annual  - 5% annual ．100% subsequent treatment ．treatment dosing and duration  - TOT: Weibull  - TOT: PFS as proxy  - TOT: Capped by PFS ．efficacy assumptions  - OS: LP/TPC KM + log-logistic  - PFS: LP/TPC 10-week KM + log-normal  - PFS: LP/TPC 37-week KM + log-logistic  - PFS: LP/TPC one-piece log-logistic ．utility  - health state utility approach  - UK tariff  - age-adjusted exclude ．societal perspective ．aEC with pMMR | | ．time horizon - 5, 30, 40, 50 years ．discount rate  - 3.5% annual  - 1.5% annual ．LYs ．price reduction  - 90% LP price  - 80% LP price  - 70% LP price ．conversion factor - 0.9 | |
| PSA | 1,000 Monte-Carlo simulations | | 1,000 Monte-Carlo simulations | | 1,000 Monte-Carlo simulations | |
| **Result** | LP is recommended. | | LP is cost-effective versus chemotherapy. | | LP is not cost-effective versus chemotherapy. | |
| Base-case result | ICER: £65,111/QALY | | ICER: SEK795,712/QALY | | ICER: NTD3,197,177/QALY | |
| DSA result^#^ | ．overall survival ．time on treatment extrapolations ．utility values | | ．overall survival  ．time on treatment | | ．time horizon ．medication cost of LP | |
| PSA result | ．mean ICER: £65,511 ．highly consistent with the base case results | | ．mean ICER: SEK 819,757/QALY ．95% chance of LP being cost-effective | | ．5.5% chance of LP being cost-effective | |
| Scenario | the most impactful scenarios | | the most impactful scenarios | | the most impactful scenarios | |
|  | scenario | ICER (QALY) | scenario | ICER (QALY) | scenario | ICER (QALY) |
|  | ．1.5% discount rate | £55,727 | ．0% discount rate | SEK629,817 | ．LY | NTD2,580,678 |
|  | ．TOT extrapolation | N/A | ．TOT of PFS as proxy | SEK997,266 | ．70% LP price | NTD2,436,180 |
|  | ．health state utility values | £71,637 |  |  | ．5-year time horizon | NTD4,772,241 |
| 95% CI, 95% confidence interval; AE, adverse event; aEC, advanced endometrial carcinoma; BNF, British National Formulary; CC, cervical cancer; ChT, chemotherapy; DSA, deterministic sensitivity analysis; ECHO, endometrial cancer health outcomes study; Emit, electronic Market Information Tool; ICER, incremental cost-effectiveness ratios; IPD, individual patient data; KM, Kaplan-Meier; LP, lenvatinib plus pembrolizumab; N/A, not applicable; NHI, National Health Insurance; NHIA, National Health Insurance Administration; NHIDB, National Health Insurance Database; NHS, National Health Service; NSCLC, non-small cell lung cancer; OS, overall survival; OWSA, one-way sensitivity analysis; PD, progressed disease; PFS, progression-free survival; pMMR, mismatch repair proficient; PSA, probabilistic sensitivity analysis; QALYs, quality-adjusted life years; RCC, renal cell carcinoma; TLV, Tandvårds- och l€akemedelsf€ormånsverket; TOT, time on treatment; TPC, treatment of physician’s choice; WTP, willingness to pay. | | | | | | |
| # Major factors that cause ICER uncertainty. | | | | | | |

References

1. National Institute for Health and Care Excellence. Pembrolizumab with lenvatinib for previously treated advanced or recurrent endometrial cancer; 2023.
2. National Institute for Health and Care Excellence. Pembrolizumab with lenvatinib for previously treated advanced, metastatic or recurrent endometrial cancer. Single Technol Appraisal 2023;2023. <https://www.nice.org.uk/guidance/ta904/evidence/appraisal-consultation-committee-papers-pdf-13070962189>, [accessed 2024 April 10]*:[ID3811], Committee Papers*.
3. Ralph L, Young K, Upadhyay N, et al. Cost effectiveness of pembrolizumab plus lenvatinib compared with chemotherapy for treating previously treated advanced endometrial cancer in Sweden. J Med Econ 2024:1-–9.
